# A Bacterial Protein Puts a New Twist on DNA Transcription

**DOI:** 10.1371/journal.pbio.0040294

**Published:** 2006-08-15

**Authors:** Mason Inman

For organisms to adapt, develop, and simply live, they must regulate hundreds to thousands of genes, making fine-tuned, precisely timed adjustments to produce the specific complement of proteins required for the occasion. For bacteria, this task falls largely to proteins called sigma factors. These small proteins associate with RNA polymerase, the enzyme that mediates gene transcription, to form a complex called the holoenzyme. The holoenzyme, guided by the sigma factor, recognizes promoter regions, which are specific DNA sequences that precede protein-coding sequences and mark the transcription start site. Sigma factors also contribute to transcription by facilitating DNA strand separation, which must occur before RNA polymerase can begin copying the DNA code. Once transcription begins, the sigma factor disengages from the RNA polymerase, becoming available for new joint ventures with different RNA polymerases.

A single sigma factor can control the expression of hundreds of genes through these partnerships, carrying out everything from basic metabolic activities to physiological responses to environmental stress (which, for bacteria, might include antibiotic therapy). Knowing how sigma factors bind to DNA is an important step in understanding how they mediate their cosmopolitan regulatory duties. Structural studies provide important clues to the nature and function of associations between sigma factors and DNA. In a new study, William Lane and Seth Darst used structural analysis techniques to determine the detailed shape of one type of sigma factor. They show that it binds to short DNA sequences using a molecular recognition method that has not been seen before in sigma factors.

Sigma factors come in two structurally unrelated families: sigma 54 and sigma 70. The sigma 54 family is associated with a diverse range of metabolic processes. The much larger sigma 70 family encompasses four groups: the Group I “primary” sigma factors facilitate metabolic and growth processes; the Group II–IV “alternative” sigma factors mediate specialized processes like sporulation and the environmental stress response. The sigma 70-type sigma factors recruit the RNA polymerase holoenzyme to bipartite promoter sequences, comprising conserved sequence elements centered about 10 and 35 base pairs upstream of the transcription start site. These so-called –10 and –35 elements are recognized by distinct structural domains of the sigma factor. Structures of one of the most studied sigma factors, a primary sigma factor called sigma-A, have been solved in previous studies. Here, Lane and Darst analyzed the –35-element-binding domain (domain 4) of an alternative Group IV sigma factor found in *Escherichia coli*, called sigma E4. Group IV sigma factors comprise the largest and most diverse set of sigma factors.

Both sigma-A4 and sigma-E4 allow RNA polymerase to bind to the –35 promoter element, but in each case the sequence is very different. In the case of sigma-E4, the sequence is GGAACTT (and others that resemble it). Previous studies showed that sigma-A4 recognizes its consensus sequence, TTGACA, through direct interactions with these six nucleotide bases. It was tempting to assume that sigma-E4 would operate in a similar manner, since the two sigma factors are similar in structure.

**Figure:**
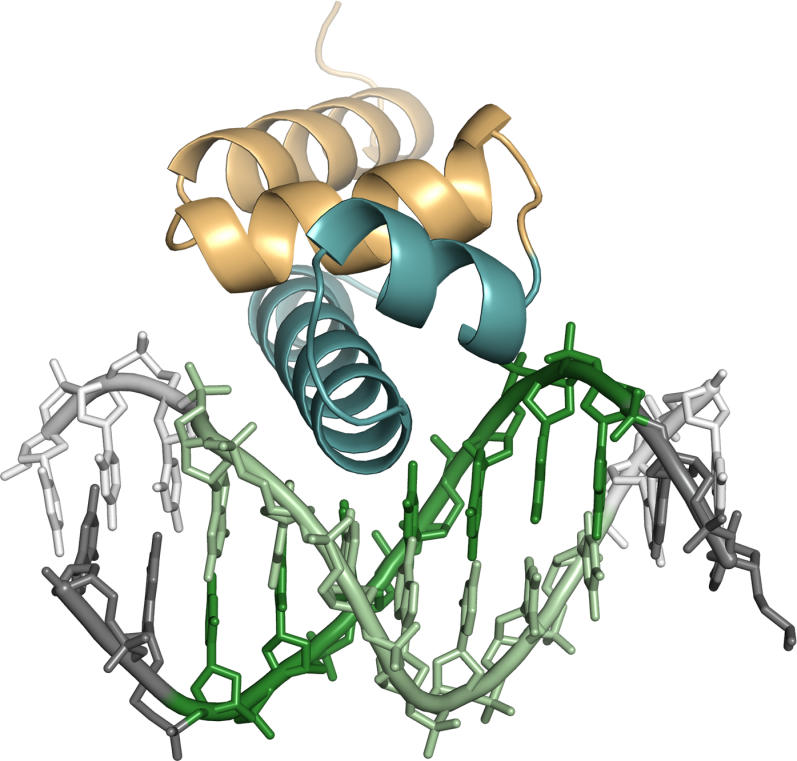
A crystal structure of domain 4 of an *E. coli* Group IV sigma factor bound to –35 element DNA identi? es a unique interaction mechanism that relies on the rigid conformation of the DNA consensus sequence

But, using X-ray crystallography, Lane and Darst showed that sigma-E4 binds its consensus sequence using a more subtle method. By determining the structure of the sigma factor bound to its consensus sequence, they found that sigma-E4 doesn’t recognize the identity of the sequences per se but the shape of the DNA helix at those sequences. While one region of the sigma factor sits deep within a groove along the double helix’s side, another region holds the promoter –35 sequence straight. The AA in the center of sigma-E4’s consensus sequence, the researchers believe, is required for the DNA to assume this shape.

Because evolution has conserved the site in these proteins that sits alongside the AA of the consensus sequence, Lane and Darst propose that this method of recognizing –35 promoter sequences may be common across the Group IV sigma factors. With further studies of the structures of sigma factors and their means of recognizing specific promoters—and thus activating specific genes—researchers can better predict the full complement of genes a given promoter will regulate, and in turn gain insight into the diverse physiological responses they help mediate.

